# Impact of Work-Related Problems on the Outcomes of Rotator Cuff Repairs: A Retrospective Comparative Study of Patients With and Without Work-Related Claims

**DOI:** 10.7759/cureus.71589

**Published:** 2024-10-16

**Authors:** Jorge H Assunção, Pedro T Da Silva, Mauro E Gracitelli, Caio Checchia, Eduardo A Malavolta

**Affiliations:** 1 Orthopaedics and Traumatology, Instituto de Ortopedia e Traumatologia, Hospital das Clínicas da Faculdade de Medicina da Universidade de São Paulo (HCFMUSP), São Paulo, BRA; 2 Orthopaedics, Instituto de Ortopedia e Traumatologia, Hospital das Clínicas da Faculdade de Medicina da Universidade de São Paulo (HCFMUSP), São Paulo, BRA; 3 Orthopaedics, Núcleo de Ombro e Cotovelo, Hospital Sírio-Libanês, São Paulo, BRA

**Keywords:** arthroscopy, orthopedic care, prognostic factors, rotator cuff tear, work-related claims

## Abstract

Background: Rotator cuff syndrome is a common cause of medical appointments and surgeries. The aim of this study is to compare the clinical outcomes of patients with work-related problems who underwent arthroscopic repair of full-thickness rotator cuff tears (RCTs) versus those without work-related or social security claims.

Methods: A retrospective cohort study comparing the outcomes of American Shoulder and Elbow Surgeons (ASES) and University of California at Los Angeles Shoulder Rating (UCLA) scores 24 months after arthroscopic repair of full-thickness RCTs of patients with and without work-related problems was performed. Patients with work-related problems were defined as those who received financial assistance from their employer or social security for more than 16 weeks before or after surgery, or those who were unable to return to work or had to change or re-adapt their job function.

Results: We evaluated 419 shoulders (411 patients), 102 shoulders with work-related claims and 317 shoulders without these claims. ASES and UCLA scores from both groups improved significantly (p < 0.001) 24 months after surgery. Patients with work-related problems had comparatively lower preoperative ASES and UCLA scores (p = 0.047 and p = 0.021, respectively) and obtained lower values after intervention for both scores, achieving 71.9 ± 18.8 on the ASES score and 28.1 ± 5.6 on the UCLA score at 24 months post-operatively. Meanwhile, patients without work-related complaints scored 82.1 ± 19 points on the ASES score and 30.1 ± 5.6 points on the UCLA score at 24 months post-operatively (p = 0.007 and p = 0.045, respectively).

Conclusion: At two-year follow-up, patients with work-related claims have significant improvement after arthroscopic repair of full-thickness RCTs by the ASES and UCLA scores. However, they have worse clinical outcomes than patients without these claims.

## Introduction

Rotator cuff syndrome is a common cause of medical care surgery [1], and among workers, it is the second leading cause of orthopedic care, behind spine-related complaints [2]. Work-related injuries cause enormous harm to employees, employers, and social security [3,4].

Rotator cuff repair leads to satisfactory clinical outcomes [5,6]. However, there is evidence that outcomes are inferior when the patients being operated have simultaneous work-related problems [4,7-9]. Lower adherence to treatment [10], psychosocial factors [11,12], and fear of re-injury [13] are possible factors related to these results.

There are a small number of published studies evaluating clinical outcomes of rotator cuff repairs in patients with work-related claims, and most of them are case series with small samples [7,14-17]. There are some comparative studies that evaluate possible confounding factors [4,9]. However, they are also composed of small samples and have no uniformity regarding surgical indications and techniques [4,9].

The aim of this study is to compare the clinical outcomes of a large series of patients undergoing arthroscopic repair of full-thickness rotator cuff tears (RCTs), divided into two groups: those with work-related claims versus those without labor or social security claims.

Our hypothesis is that patients with work-related claims have worse clinical outcomes than patients without these claims after arthroscopic repair of full-thickness RCTs.

## Materials and methods

Design, location, and dates

This is an institutional ethical committee-approved, retrospective cohort study, with prospectively collected data, comparing functional outcomes between patients with and without work-related problems during the treatment period. Patients with work-related problems were defined as those who received financial assistance from their employer or social security for more than 16 weeks before or after surgery, or those who were unable to return to work or had to change or re-adapt their job function. Surgeries were performed between January 1st, 2016, and April 30th, 2021, in a single hospital, by three different surgeons, with 15 to 17 years of experience.

Eligibility criteria

During the time frame of the study, every patient under 65 years of age who underwent arthroscopic repair of full-thickness RCTs, and had a pre-operative MRI, (without the use of intra-articular contrast) on a 1.5T or higher magnet, was included in the study. All of them had failed non-operative treatment prior to surgery (Patients underwent physiotherapeutic treatment and the use of analgesic and anti-inflammatory medications for at least 6 to 12 weeks without success). Criteria for non-inclusion were irreparable or partially repaired RCTs; isolated subscapularis tears; rotator cuff arthropathy; moderate or severe glenohumeral arthrosis [18]; previous shoulder surgery; fatty degeneration of any of the rotator cuff muscles ≥ stage 3 [19]; lack of pre-operative assessment; post-operative follow-up ≤ 24 months.

Outcomes

The primary outcomes were *American Shoulder and Elbow Surgeons* (ASES) [20] and *University of California at Los Angeles Shoulder Rating *(UCLA) scores [21] at 24 months follow-up. Secondary outcomes were the gains obtained for these scores 24 months after surgery (post-minus pre-operative scores).

The ASES score is a mixed-outcome reporting tool, meaning it consists of a physician-rated and patient-rated questionnaire. The patient-rated questionnaire focuses on joint pain, instability and activities of daily living. The total score is weighted 50% for pain and 50% for function. The pain score is calculated by subtracting the VAS from 10 and multiplying it by 5. The 10 functional questions are scored on a 4-point scale (0-3) with a maximum functional score of 30. The raw functional score is then multiplied by 5/3 to make the maximum functional score out of 50 possible points. The pain and function scores are then added together to obtain the final ASES score (out of 100). Higher scores correlate to better outcomes.

The UCLA score is a patient-based survey that evaluates shoulder conditions. It's a combination of objective and subjective measures that both the patient and surgeon complete. Muscle strength, shoulder active forward flexion, pain, satisfaction, and patient function are measured. The UCLA shoulder score has a total score range of 2 to 35, with higher scores indicating better function. 

Variables analyzed

Patient-related variables analyzed are age, sex, smoking, diabetes, arterial hypertension, hypothyroidism, rheumatological diseases, previous shoulder infiltration, and previous trauma to the affected shoulder.

Tear-related variables include supraspinatus retraction (<30mm or ≥30mm), presence of subscapularis tear, long head of the biceps (LHB) injury, LHB instability, and degree of fatty infiltration (subscapularis, supraspinatus and infraspinatus).

Surgery-related variables are subscapularis repair, acromioplasty, distal clavicle resection, and biceps procedure.

All tear-related variables, except for fatty degeneration, were assessed by arthroscopic inspection and reported by the surgeon. Clinical evaluation using ASES and UCLA scores was performed one week before surgery and 24 months after it and was collected by a research assistant who was not involved in the study.

Magnetic resonance imaging

Patients underwent pre-operative MRI on 1.5 Tesla (or higher) magnets, without the use of intra-articular or intravenous paramagnetic contrast, no more than six months before surgery.

Surgical procedure

Surgeries were performed under general anesthesia with an interscalene block. Patients were positioned in either beach chair or lateral decubitus positions, depending on the surgeon's preference, and conventional portals were used. Inspection was performed in a standardized manner, with a 30° optic through the posterior portal. Using a probe, the LHB was palpated and mobilized, looking for signs of instability (using the "ramp test" [22]), as well as lesions in its substance or insertion.

The LHB was addressed when it presented subluxation or dislocation, partial lesions involving more than 50% of its thickness, or in the presence of type 2, 3 or 4 superior labrum lesions. For patients aged 60 years or older, tenotomy was preferred. Tenodesis was performed in athletes, younger patients, or those with BMI < 25, regardless of age. A subscapularis tendon tear was repaired if it was full-thickness or if a partial tear had ≥ 5 mm of footprint exposure. Repair of the posterosuperior tear was performed using a single-row technique with 5 mm anchors, after greater tuberosity debridement. Acromioplasty was performed in patients with Bigliani type 2 or 3 acromions [23], and distal clavicle resection in patients with symptomatic arthritis. After surgery, patients remained immobilized in a sling for six weeks. Passive shoulder movements started after two weeks, and active movements after the sling was removed. Strengthening started at 12 weeks.

Statistical analysis

Continuous variables were assessed for normality using the Shapiro-Wilk test and for homogeneity using the Levene test. Categorical variables were presented in absolute and percentage values. Continuous variables were presented as mean and standard deviation. The general characteristics of the sample were compared between groups using the chi-square or Fisher's test (for categorical variables) or the T-test (for continuous variables).

Intra- and inter-group comparisons between pre- and post-operative results, according to ASES and UCLA scores, were performed by the T-test. The proportion of patients in both groups who reached the Minimal Clinically Important Difference (MCID) for ASES and UCLA scores was also assessed [24].

All variables with p < 0.1 would be subject to a multiple linear regression analysis to identify confounding factors in the final outcome. Data analysis was performed using IBM SPSS Statistics for Windows, Version 21 (Released 2012; IBM Corp., Armonk, New York, United States), with a significance level of 5%.

## Results

In the time frame of this study, 743 rotator cuff repairs were performed, 63 of which were open repairs and 55 were partial tears, totaling 625 arthroscopies for full-thickness tears. Of these, 206 were excluded, because they consisted of tear debridement (n = 10), isolated subscapularis repair (n = 14), partial repairs (n = 49), patients over 65 years of age (n = 98), fatty degeneration of one or more rotator cuff muscle ≥ Goutallier stage 3 (n = 20) or those without pre-operative clinical information (n = 15). Therefore, the analyzed sample consisted of 419 shoulders (411 patients), 102 of which (102 patients) had work-related problems (mean age = 53.7 years) and 317 shoulders (309 patients) without work-related complaints (mean age = 54.3 years, p = 0.367).

Comparison of variables regarding baseline characteristics of patients showed no difference concerning distribution by sex (p = 0.443), traumatic etiology (p = 0.351), previous subacromial injection (p = 0.906), and clinical comorbidities (Table 1).

**Table 1:** Baseline characteristics of 411 patients The general characteristics of the sample were compared between groups using the chi-square or Fisher's test (for categorical variables) or the T-test (for continuous variables).

	Work-related claims				
	Yes (n=102)		No (n=309)		p
Age, years (mean (SD))	53.7	7.9	54.2	7.3	0.367
Sex (n (%))					
Men	52	51.0	142	46.0	0.443
Women	50	49.0	167	54.0	
Hypothyroidism (n (%))					
Yes	8	7.8	32	10.4	0.582
No	94	92.2	277	89.6	
Diabetes (n (%))					
Yes	8	7.8	39	12.6	0.256
No	94	92.2	270	87.4	
Systemic arterial hypertension (n (%))					
Yes	21	20.6	90	29.1	0.120
No	81	79.4	219	70.9	
Rheumatologic diseases (n (%))					
Yes	3	2.9	17	5.5	0.437
No	99	97.1	292	94.5	
Smoking (n (%))					
Yes	16	15.7	29	9.4	0.113
No	86	84.3	280	90.6	
Previous injection (n (%))					
Yes	15	14.7	42	13.6	0.906
No	87	85.3	267	86.4	
Traumatic tear (n (%))					
Yes	22	21.6	52	16.8	0.351
No	80	78.4	257	83.2	

Comparisons of baseline variables related to the tears and procedures also showed no statistically significant difference in all analyses. Data can be seen in Table 2.

**Table 2:** Baseline characteristics of 419 shoulders for rotator cuff tear and procedures The general characteristics of the sample were compared between groups using the chi-square or Fisher's test. LHBT: Long head of the biceps tendon

	Work-related claims				
	Yes (n=102)		No (n=317)		p
Supraspinatus tear retraction (n (%))					
< 30mm	78	76.5	229	72.2	0.477
≥ 30mm	24	23.5	88	27.8	
Subscapularis tear (n (%))					
Yes	41	40.2	157	49.5	0.127
No	61	59.8	160	50.5	
Infraspinatus tear (n (%))					
Yes	21	20.6	80	25.2	0.411
No	81	79.4	237	74.8	
LHBT lesion (n (%))					
Complete	3	2.9	24	7.6	0.272
≥ 50%	14	13.7	30	9.7	
< 50%	21	20.6	64	20.9	
No	64	62.7	199	64.8	
LHBT stability (n (%))					
Stable	73	73.7	204	69.6	0.520
Unstable	18	18.2	54	18.4	
Dislocated	8	8.1	35	12.0	
Goutallier's classification supraspinatus (n (%))					
Grade 0	36	35.3	107	33.8	0.818
Grade 1	48	47.1	160	50.5	
Grade 2	18	17.6	50	15.8	
Goutallier's classification infraspinatus (n (%))					
Grade 0	47	46.1	145	45.7	0.299
Grade 1	39	38.2	139	43.8	
Grade 2	16	15.7	33	10.4	
Goutallier's classification subscapularis (n (%))					
Grade 0	57	55.9	196	61.9	0.502
Grade 1	33	32.6	93	29.3	
Grade 2	12	11.8	28	8.8	
Acromioplasty [n (%)]					
Yes	78	76.4	241	76.0	0.967
No	24	23.6	76	24.0	
Distal clavicle resection (n (%))					
Yes	3	2.9	10	3.3	0.999
No	99	97.1	307	96.7	
Subscapularis repair (n (%))					
Yes	31	30.4	127	40.1	0.102
No	71	69.6	190	59.9	
LHBT procedure (n (%))					
Tenotomy	21	20.6	58	18.3	0.122
Tenodesis	27	26.5	119	37.5	
None	54	52.9	140	44.1	

The procedure yielded significant improvements in both groups according to ASES and UCLA scores (p<0.001). However, patients with work-related claims had lower pre-operative ASES and UCLA scores (p = 0.047 and p = 0.021, respectively) at the final follow-up (24 months). They obtained 71.9 ± 18.8 points on the ASES score and 28.1 ± 5.6 on the UCLA score, while patients in the other group reached an ASES score of 82.1 ± 19 points and a UCLA score of 30.1 ± 5.6 (p = 0.007 and p = 0.045, respectively) (Table 3).

**Table 3:** Pre- and post-operative results of 419 shoulders by work-related claims *Difference between post-operative and pre-operative scores The ASES score ranges from 0 to 100, with 0 being the lowest level of function and 100 being the highest level of function. The UCLA score ranges from 2 to 35, with 2 being the lowest level of function and 35 being the highest level of function. Group comparisons between pre- and post-operative results, according to ASES and UCLA scores, were performed by the T-test. UCLA: University of California at Los Angeles Shoulder Rating; ASES: American Shoulder and Elbow Surgeons

	Work-related claims				
	Yes (n=102)		No (n=317)		p
	Mean	SD	Mean	SD	
ASES score					
Pre-operative	33.1	21.7	41.4	21.3	0.047
24 months post-operative	71.9	18.8	82.1	19.0	0.007
Gain*	39.1	21.2	41.6	23.8	0.213
UCLA score					
Pre-operative	13.6	4.1	15.6	5.5	0.021
24 months post-operative	28.1	5.6	30.1	5.6	0.045
Gain*	14.7	6.5	15.4	6.1	0.380

There was no statistical difference between groups regarding differences in pre- and post-operative ASES and UCLA scores (p = 0.213 and p = 0.380); both groups improved by approximately 40 points on the ASES score and 15 points on the UCLA score. However, among those patients with work-related problems, 20 (19.6%) did not achieve MCID at the final follow-up for the ASES score and 11 (10.7%), for the UCLA score. Among patients without work-related complaints, 31 (9.8%) did not achieve MCID for the ASES score and 14 (4.4%) for the UCLA score (p=0.006 and p =0.038, respectively).

## Discussion

Our results demonstrated that 24 months post-operatively, arthroscopic repair of full-thickness RCTs yielded worse functional outcomes in patients with work-related claims than in patients without work-related claims. This finding is similar to that of Holtby et al. [4], who conducted a case-control study comparing results one year after surgery. They evaluated WORC [25], ASES [20], and Constant [26] scores, as well as forward flexion strength, and found statistically significant differences between groups regarding all scores. Henn et al. also found statistical differences one year after surgery by the Simple Shoulder Test, DASH, and Short Form-36 scores between both groups, with worse results in the group of patients with work-related claims [9].

In our study, patients with work-related problems also had lower pre-operative scores compared to the other group, a finding similar to those of Holtby et al. [4] and Henn et al. [9].

Both our groups obtained significant improvements at the final follow-up, with gains of approximately 40 points on the ASES score and 15 on the UCLA score. We found no statistical difference in gains between groups. Holtby et al. reported different results from ours, where patients with work-related problems achieved smaller gains on the ASES, WORC, and Constant scores compared to the other group [4]. Patients with work-related complaints had a gain of only 20 points on the ASES score, while the other group gained 31 points. However, both groups benefited from the treatment. Henn et al. found statistically significant improvements in SST, DASH score, and VAS for pain and shoulder function for both groups [9]. The group of patients with work-related problems improved less than the other group for every outcome variable analyzed, but this difference was statistically significant only for the DASH score and the VAS for pain and shoulder function.

In our study, despite the fact that both groups obtained similar mean gains to both outcome variables, the number and the percentage of patients who did not reach MCID at final follow-up was significantly higher in the group with work-related claims for the ASES (19.6%) and UCLA scores (10.7%). Those percentages were statistically lower in the other group, which were 9.8% for the ASES score and 4.4% for the UCLA score.

Our eligibility criteria included only patients under 65 years of age since the number of workers after this age is supposedly lower due to retirement. Another reason is that an absence of an age cutoff could result in unbalanced groups, possibly with many more older individuals in the group of patients without work-related complaints. These older individuals have more comorbidities, larger tears, and greater fatty infiltration [27], generating possible confounding factors. Both groups in our study had similar baseline characteristics regarding age, sex, comorbidities, and tear patterns.

Kim et al., in a retrospective comparative case-control study, observed that patients with work-related problems had worse ASES and UCLA scores one year after surgery [7]. However, at the two-year post-operative follow-up (after termination of social security benefits), both groups had similar scores. In our study, on the other hand, we observed that the differences in scores remained two years after the surgical procedure. However, we do not know how many of our patients were still receiving some social security benefits, had not returned to work, or were adapted to a different labor function at the last follow-up, which is a limitation of this study.

Several factors, such as lower adherence to treatment [10], psychosocial factors [11,12], job demands, secondary gain, and fear of a new injury [13], have been postulated to explain the differences observed in the results between patients with and without work-related complaints. Cuff and Pupello analyzed whether patient adherence to a post-operative protocol affected the outcomes of patients with social security problems [10]. The authors concluded that patients with workers' compensation claims showed a high rate of post-operative dropout (52% versus 4%). Patients with work-related accidents, who showed no evidence of non-compliance to post-operative protocols, had significant improvement and more favorable outcomes than those with work-related accidents who did not adhere. In our study, all patients followed the same rehabilitation protocol and we did not have a control of adherence to it.

Our study has other limitations. It is a retrospective cohort study, with the biases inherent to this design. We did not perform post-operative MRI, so it was not possible to analyze the structural integrity of repairs, similar to all other studies on this subject. However, it is known that functional outcomes do not directly correlate to cuff integrity [28]. A lack of evaluation of the job position of our patients, as well as whether it involved repetitive strain to their upper limbs, is another limitation as this may be an unverified confounding factor. We did not use scales in the study that could assess psychosocial factors and patient resilience, which would help evaluate possible secondary gains and inferior clinical outcomes in patients receiving social security benefits.

The highlights of our study are as follows. We were able to analyze a large number of patients, greater than other comparative studies on this subject [4,7,9]. Measurement biases were minimal, given a standardized clinical evaluation at 24 months post-operatively, carried out by a research assistant who did not participate in the study. We included only repairs of full-thickness RCTs, unlike other studies, which included different pathologies related to rotator cuff syndrome [4], as well as additional different surgical procedures, such as open surgeries or only subacromial decompression [4,9].

## Conclusions

At the two-year follow-up, patients with work-related claims have significant improvement after arthroscopic repair of full-thickness rotator cuff tears, obtaining gains in functionality similar to patients without work-related complaints, by the ASES and UCLA scores. However, patients with work-related claims have worse clinical outcomes at 24 months post-operatively than patients without these claims, and a greater proportion of patients do not reach the minimal clinically important difference at the final follow-up for ASES and UCLA scores.
